# Comparison of Short-Sprint and Heavy Strength Training on Cycling Performance

**DOI:** 10.3389/fphys.2019.01132

**Published:** 2019-08-28

**Authors:** Morten Kristoffersen, Øyvind Sandbakk, Bent R. Rønnestad, Hilde Gundersen

**Affiliations:** ^1^Department of Sport, Food and Natural Sciences, Western Norway University of Applied Sciences, Bergen, Norway; ^2^Centre for Elite Sports Research, Department of Neuroscience and Movement Science, Norwegian University of Science and Technology, Trondheim, Norway; ^3^Section for Sport Science, Inland Norway University of Applied Sciences, Lillehammer, Norway

**Keywords:** road cycling, short-sprint training, strength training, concurrent training, power output, trained subjects

## Abstract

**Purpose:**

To compare the effects of short-sprint training (SST) and heavy-strength training (HST) following a 4-week strength-training period on sprint and endurance capacities in well-trained cyclists.

**Methods:**

Twenty-eight competitive cyclists (age 29 ± 6 years) with maximal oxygen uptake (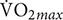) of 61.1 ± 5.9 mL⋅min^–1^⋅kg^–1^ participated. After a 4-weeks preparation strength-training period, the participants were randomized to add either HST or SST to their usual endurance training for the subsequent 6 weeks. Body composition, 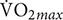 and power output at blood lactate concentration ([La^–^]) of 4 mmol⋅L^–1^, as well as a 100 min cycling test including 6 and 30-s sprints, 60 min cycling at [La^–^] of 2 mmol⋅L^–1^ and 5-min all-out cycling were performed before the 4-week preparation strength-training period, and before and after the 6-week intervention period. In addition, 1 repetition maximum (RM) in half-squat and 55-m maximal sprints on the cyclists’ own bikes were measured before and after the 6-week intervention.

**Results:**

SST was superior to HST in 6-s sprint performance, both in a fresh state (4.7 ± 2.6% vs. 1.1 ± 3.5%) and after prolong cycling (6.1 ± 1.8% vs. 1.8 ± 4.2%), in 30-s sprint (3.7 ± 2.8% vs. 1.3 ± 2.5%) and in 55-m seated sprint on own bike (4.3 ± 2.1% vs. 0.2 ± 1.8%) (all *p* < 0.002). HST induced a larger 1RM improvement in the half-squat test than SST (9.3 ± 3.6% vs. −3.9 ± 3.8%; *p* < 0.001). No group differences were revealed in the 5-min all-out test, 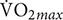, power output at 4 mmol⋅L^–1^ [La^–^], or in gross efficiency.

**Conclusion:**

SST led to a greater increase in average and peak power output on all sprint tests compared to HST, whereas HST led to a greater increase in maximal strength. No group differences were found in relative changes in endurance capacities. Altogether, our results show a high degree of specificity in the adaptations of both SST and HST.

## Introduction

Road cycling is a demanding endurance sport, with performance mainly determined by maximal aerobic power, the ability to utilize a large fraction of maximal aerobic power over long-lasting competitions and cycling efficiency (Jeukendrup et al., 2000; Faria et al., 2005). In addition, the ability to perform breakaways, to close gaps, and to sprint fast in the finish-sprint are important to win races (Faria et al., 2005). Although endurance training is undoubtedly the most important component in cyclists’ training regimen, training with the goal to increase the capacity to produce high-power output for a relatively short duration through heavy strength and sprint training could be beneficial. In fact, especially during the pre-season, many competing cyclists include strength training in addition to the usual endurance training with the goal to increase the capacity to produce high-power output. A strength-training period usually starts with a preparation period focusing on building basic strength capacity through high volume and medium load, followed by a period with heavy strength training (HST) to increase maximum strength through lower volume and higher load. Finally, there is a sport specific high power strength period focusing on maximal power development (Kraemer and Ratamess, 2004; Fleck, 2011). It has been shown positive effects of HST on various factors related to cycling performance, without any negative interference effects on the endurance capacity (Koninckx et al., 2010; Ronnestad et al., 2010a, b, 2011, 2015a, 2016; Aagaard et al., 2011; Ronnestad and Mujika, 2014; Vikmoen et al., 2016). To the best of our knowledge, the effect on cycling performance of going directly from the basic strength-training phase to the sport specific maximal power development phase has not been compared with the traditional approach of moving from the basic strength phase to the maximum strength phase.

Although many road cyclists regularly carry out short-sprint training (SST) in addition to the usual endurance training during the pre-season, only a few studies have examined the effects of this training method. Specifically, two previous studies found that only 2 weeks of SST, involving 6-s sprint intervals, improved peak power output (PPO) and 10 km time trail cycling performance in triathletes (Jakeman et al., 2012) and in physically active men (Lloyd Jones et al., 2017). Another study conducted with trained students found that 7 weeks of SST, performed as 5-s sprint intervals, improved PPO and total work during 30-s all-out cycling (Linossier et al., 1993). Consequently, both HST and SST are common methods in competitive cyclists. However, to date, no studies have compared the effects of HST and SST during the pre-season on sprint and endurance capacities. Therefore, the main aim of the present study was to compare the effects of 6-week HST or SST after a 4-week preparation strength period on sprint and endurance capacities in trained cyclists. In addition, we compared whether the possible effects from these types of training could be transferred to sprinting ability on the cyclists’ own bike. We hypothesized that a larger improvement in sprint performance would occur after SST (as compared to HST), while we expected no differences between SST and HST in endurance capacities.

## Materials and Methods

### Participants

Thirty-two participants (28 men and 4 women) were included in the study, but due to injury and illness, four participants withdrew. Thus, in total, 26 men and 2 women (age 29.6 ± 0.6 years; height 183 ± 7 cm, weight 79.3 ± 9.0 kg) fulfilling at least two of Jeukendrup’s criteria for trained road cyclists completed the study (Jeukendrup et al., 2000).

The Regional Committee for Medical and Health Research Ethics in Western Norway evaluated the study to ensure that it did not include any medical or health-related ethical concerns. The study was then approved by The Norwegian Data Protection Authority. All participants gave their written, informed consent to participate in the study, which was completed according to the Declarations of Helsinki.

### Experimental Design

**FIGURE 1:**
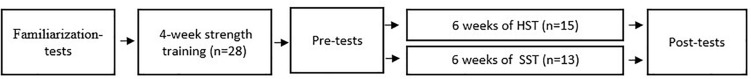
An overview of the experimental design. HST, heavy strength training; SST, short-sprint training.

A pre-post design was used in this study (Figure 1). Participants were tested before and after a 6-week training intervention. After a 4 weeks preparation strength period, participants were randomized either to implement SST (*n* = 16) or HST (*n* = 16) two times a week (in addition to their regular endurance training). All cyclists (except three from the SST group and one from the HST group) completed at least 10 of the 12 SST or HST sessions during the 6-week training intervention, and had done at least 6 out of 8 HST sessions in the 4-week preparation strength-training period. Preliminary analysis showed no differences between groups for any of the variables before start of the 6-week intervention (Table 2; all *p* > 0.05).

### Strength-Training Protocol

A daily undulating periodization HST program (Rhea et al., 2002) based on previous studies reporting significant improvement in strength and cycling performance in well-trained cyclists was used (Ronnestad et al., 2010b, 2015a, 2016). The strength exercises included half-squat in a Smith machine, leg press with one foot at a time, one-legged hip flexion, and toe raise (Ronnestad et al., 2015b). The HST was conducted with the intention of maximally accelerating the load during the concentric phase, while the eccentric phase was performed more slowly.

In the 4-week preparation strength training period, before the intervention, all participants performed 3 sets of 10RM in the first weekly session and 3 sets of 6RM in the second weekly session during the three first weeks, and 3 sets of 8RM and 3 sets of 5RM the last week. During the 6-week intervention, the participants in the HST group performed 3 sets of 8RM in the first weekly session and 3 sets of 5RM in the second weekly session for 2 weeks, and 3 sets of 6RM and 3 sets of 4RM during the four following weeks. Rest between sets and exercises was 2 min. To ensure proper technique and load, an investigator supervised all participants during the first week in the preparation period and all participants in the HST group at the first session in the 6-week intervention period.

The participants in the HST group were encouraged to continuously increase their RM loads throughout the intervention period and to record load (kg), repetitions, and sets performed during each session into an electronic form after each session. They were also encouraged to complete the workouts with another participant from the HST group.

### Short-Sprint Training Protocol

The SST program was based on a recent study conducted in our laboratory (Kristoffersen et al., 2018). All sprints in the SST sessions were conducted using an air-braked cycle ergometer (WattBike Ltd., Nottingham, United Kingdom) in a seated position, from a standstill start, with the preferred leg chosen by the participant. All the participants got the cycle ergometers individually adjusted before entering the study. Moreover, the pedaling resistance applied to the sprints was individually adjusted in the first SST session, where each participant performed at least three 6-s seated sprints at different resistance levels to ensure that the participant achieved the highest possible power output at a cadence of 110–120 rpm (Hopker et al., 2010; Herbert et al., 2015). The SST session consisted of 15 min warm-up, followed by three sets of four intervals with maximum effort (separated by 2 min of active recovery between intervals), and 5 min active recovery between sets of cycling at 70% maximal heart rate (HR_*max*_). To reflect practise, a daily undulating periodization SST program with progressive overload by increasing the duration of the sprint intervals every second week was used. During the first 2 weeks, the first and second weekly sessions consisted of 4- and 6-s sprints, respectively. The subsequent 2 weeks consisted of 7- and 5-s sprints, and the final 2 weeks consisted of 8- and 6-s sprints. All sprint sessions were monitored and transferred to the Training Peaks diary through the participants’ bike computers.

### Testing Procedures

**FIGURE 2:**
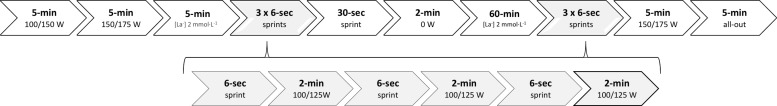
An overview of time and load in the different phases of the 100 min cycling test. W, watt (female/male); [La^–^] 2 mmol⋅L^–1^, load at blood lactate concentration of 2 mmol⋅L^–1^ measured in blood lactate profile test performed before the first test period. 6-s sprints and 2 min rest between sprints performed in fresh state and after prolonged cycling are marked in gray.

Each of the test periods, including the familiarization tests before the 4-week preparation strength training period, consisted of two separate test days: Day (1) Measurement of body composition, and a stepwise incremental cycle test to determine the power output at [La^–^] of 2 mmol⋅L^–1^, followed by a 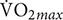 test. Day (2) A 100-min cycling test, including 6- and 30-s maximal sprints, and a 5-min all-out test (Figure 2). In addition, a 1RM in half-squat and 55-m maximal sprint on the cyclists’ own bikes were included on a third test day before and after the 6-week intervention.

All participants were instructed to abstain from strenuous exercise for 48 h before the tests, and to consume the same type of meal before each test day. There was a minimum of 48 h between test days, and all tests were conducted at the same time of day to avoid any possible effects of the circadian rhythm. All tests in the laboratory (day 1 and 2) were performed under uniform environmental conditions (17–19°C) on the same electromagnetic bike (Lode Excalibur Sport, Groningen, Netherlands), which was adjusted according to each cyclist’s preferences. On day 3, the 55-m sprint tests were performed under uniform environmental conditions (11°C) in an indoor garage.

Test procedures for the body composition (bioelectrical impedance) analysis, the incremental lactate profile test and the 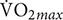 test conducted in the present study are described in detail in a previous study (Kristoffersen et al., 2018). The remaining tests are described in detail below.

### 100-min Test

The 100-min cycling test protocol was completed using the Lode Ergometry Manager (LEM 10 Software, Germany), where power output was continuously registered throughout the entire test (Figure 2).

Each participant performed the familiarization-, pre- and post-tests with the same power output in each submaximal exercise phase. 6- and 30-s sprints were performed in a Wingate mode. The load was calculated from the participant’s total body mass at baseline, and set to 0.8 (30-s) and 1.0 (6-s) Nm⋅kg^–1^ body mass in men and to 0.8 (6-s) and 0.77 (30-s) Nm⋅kg^–1^ body mass in women. The power output at [La^–^] of 2 mmol⋅L^–1^ was determined with linear regression between the nearest data points below and above 2 mmol⋅L^–1^ during the first lactate profile test at familiarization and this absolute value was used as the 2 mmol⋅L^–1^ workload during all subsequent tests.

The 5-min and 60-min phases at [La^–^] of 2 mmol⋅L^–1^ were performed with the ergometer in a cadence-independent mode. All sprints were completed seated, starting with a cadence of 50–60 rpm. The 6-s sprints were separated by 2 min of active recovery (125 W in men and 100 W in women), with a 2-min recovery without load after the 30-s sprint. The best 6-s sprint before and after prolonged cycling was used in the statistical analyses. The 5-min all-out cycling was performed with the ergometer in cadence-dependent mode (linear mode), where the power output increases with increasing cadence according to the following formula: W = *L* × (rpm)^2^, where W is the power output, rpm is the cadence, and the constant (*L*) determines the electronic gearing of the system. Here, it was set to 0.042 in men and 0.035 in women.

To calculate gross efficiency (GE) in fresh state (i.e., after 5 min cycling at 125 W in men and 100 W in women, followed by 5 min at 175 W and 150 W, respectively) and after prolonged cycling, *VO*_2_ and respiratory exchange ratio (RER) were measured between the 2nd and 4th minutes in the phase before the 6-s sprint in fresh state, and between the 50th and 53rd minutes in the 60-min phase (Figure 2). Expired gases were sampled continuously, and average values for every 30 sec were used in the calculations. 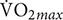 and RER were measured using a computerized metabolic system with a mixing chamber (Oxycon Pro, Erich Jaeger, Hoechberg). The work rate was then divided by the metabolic rate to calculate GE (Skovereng et al., 2018). The participants were allowed to consume sports drinks in order to maintain fluid balance and to mimic race conditions. Volume and time of consumption were noted during the first test, and the same procedure was repeated in the following tests.

### 1RM in Half Squat

1RM in half squat was measured in a Smith machine (TKO, Houston, United States). The test was preceded by a 15-min warm-up on a cycle ergometer (WattBike Ltd., Nottingham, United Kingdom). Following the warm-up, the participants performed a standardized protocol, consisting of three sets with gradually increasing load and decreasing number of repetitions. The depth of the squat was set to approximately 90°. The squat depth and the feet placement were monitored and marked to ensure similar positioning at each test (Ronnestad, 2009). The first 1RM attempt was performed with a load approximately 5 kg below the predicted 1RM. After each successful attempt, the load was increased by 2.5–5 kg.

### 55-m Sprint

Ten participants from the HST group and eight participants from the SST group performed the 55-m sprint test. Testing was performed on the participants’ own bikes, and the participants were instructed to use the same bike with the same setting in each test. Before testing, the participants performed a 15-min warm-up on an ergometer with the same load as the first 15 min in the 100-min test. All participants performed 3 seated and 3 standing sprints, with 2-min recoveries between each. Before the beginning of each attempt, the participant clipped her/his pedal, and an experimenter then maintained the participants starting position. The test was carried out with a static start, and the participants started at a verbal command. Times were measured using wireless timing gates (Muscle Lab, Ergotest Technology, Langesund, Norway). The best 55-m times in seated and standing sprints were used in the subsequent analyses.

### Training Load and Intensity

All training during the 4-week strength training period, and during the 6-week intervention period was monitored and transferred into each participants’ training diary account (Training Peak, Peaksware LLC, Lafayette, CO, United States) and analyzed with the analyzing program WKO4 (Peaksware LLC, Lafayette, CO, United States). There were no difference between the SST and HST group in neither weekly logged training duration (4-week strength training period: 12.6 ± 7.6 h vs. 11.8 ± 5.8 h, intervention period: 14.4 ± 8.6 h vs. 14.1 ± 8.4 h, respectively), nor in training intensity distribution.

### Statistical Analyses

The Statistical Products of Service Solution package (SPSS Statistics, version 24) was used for all statistical analyses. The preliminary analysis (Kolmogorov–Smirnov) showed that our data did not deviate from a normal distribution for any the considered variables. All descriptive data in text and tables are shown as mean ± standard deviation (SD). For all participants pooled, a dependent *t*-test was performed to measure the changes from the familiarization test to the pre-test. Thereafter, a 2 × 2 repeated measures ANOVA analyses were employed, with type of training (two levels: pre-test and post-test) as a within–subjects factor, and group (two levels: SST and HST) as a between-subjects factor. Independent-samples *t*-tests were used to evaluate between group differences in changes. Pearson correlation analysis was used to investigate the relationship between changes in APO in the 6-s sprint in fresh state and changes in time during the 55-m seated sprint test for each group. We used a Bonferroni correction for all pairwise comparisons in order to prevent Type I error. The corrected/adjusted *p*-value was obtained by dividing the original α-value by the number of analyses on the dependent variable. For the rest of the analysis a *p*-value of ≤0.05 was considered statistically significant. The magnitude of changes was also expressed as standardized mean differences (effect size, ES) (Cohen, 1988). ES values between 0.2 and 0.49 indicated a small effect, between 0.5 and 0.79 indicated a moderate effect and 0.8 and above indicated a large effect.

## Results

From the familiarization-test to the pre-test for all participants pooled (*n* = 28), there was a 4.2 ± 5.6% increase in PPO (*p* = 0.001) and 3.4 ± 5.1% in APO (*p* = 0.001) in the 6-s sprint test in fresh state. Additionally, all participants increased PPO by 3.8 ± 7.5% (*p* = 0.007) in the 30-s sprint test from the familiarization-test to the pre-test. No differences was found in other measurements.

### Sprint Performance

In 6-s sprint in fresh state, the SST group increased PPO (*p* = 0.001) and APO (*p* = 0.001) from pre- to post test. No significant changes were observed in the HST group in PPO (*p* = 0.289) and APO (*p* = 0.158) (Table 1). In the 6-s sprints after prolonged cycling, the SST group increased PPO (*p* = 0.001) and APO (*p* = 0.001) from pre- to post test. A significant change was observed in PPO in the HST group (*p* = 0.030) but not in APO (*p* = 0.057) (Table 1.) In addition, a significant difference was seen in change between groups from pre- to post-test in PPO and APO both in fresh state (*p* = 0.023, ES = 0.93 and *p* = 0.006, ES = 1.16, respectively) and after prolonged cycling (*p* = 0.001, ES = 1.52 and *p* = 0.003, ES = 1.42, respectively) (Table 1).

**TABLE 1:** An overview of the results obtained in the 6 and 30-s sprints, and the 5-min all-out test, measured pre and post 6-weeks of short-sprint training (SST) or heavy strength training (HST).

	**SST group**			**HST group**		
	**pre**	**post**	**% change**	**pre**	**post**	**% change**
**6-s sprint:**						
Fresh state						
PPO (W⋅kg^–1^)	17.8 ± 2.5	18.7 ± 2.5^∗^	5.6 ± 3.5	17.1 ± 2.3	17.3 ± 2.5	1.9 ± 5.8^#^
APO (W⋅kg^–1^)	14.9 ± 1.8	15.6 ± 1.8^∗^	4.7 ± 2.6	14.5 ± 1.6	14.7 ± 1.7	1.1 ± 3.5^#^
After prolonged cycling:						
PPO (W⋅kg^–1^)	17.0 ± 2.7	18.2 ± 2.8^∗^	7.2 ± 3.6	16.6 ± 2.7	17.0 ± 2.6^∗^	2.3 ± 3.6^#^
APO (W⋅kg^–1^)	14.4 ± 1.9	15.3 ± 2.0^∗^	6.1 ± 1.8	14.5 ± 1.6	14.7 ± 1.8	1.8 ± 4.2^#^
**30-s sprint:**						
PPO (W⋅kg^–1^)	15.8 ± 2.2	17.0 ± 2.30^∗^	8.0 ± 3.8	15.2 ± 2.3	15.6 ± 2.5	2.6 ± 5.9^#^
APO (W⋅kg^–1^)	9.6 ± 0.9	9.9 ± 0.9^∗^	3.7 ± 2.8	9.3 ± 0.7	9.4 ± 0.7	1.3 ± 2.5^#^
**5-min all-out**						
APO (W⋅kg^–1^)	4.7 ± 0.5	5.0 ± 0.5^∗^	5.7 ± 5.0	4.6 ± 0.6	4.8 ± 0.5^∗^	3.3 ± 4.4
**55-m sprint**						
Seated position (sec)	7.55 ± 0.40	7.21 ± 0.28^∗^	−4.3 ± 2.1	7.47 ± 0.23	7.46 ± 0.28	−0.2 ± 1.8^#^
Standing position (sec)	7.11 ± 0.33	7.06 ± 0.34	−0.7 ± 2.6	7.12 ± 0.33	7.10 ± 0.33	−0.4 ± 1.5

*Values are mean ± SD. ^∗^Differences within groups (*p* < 0.05); ^#^Difference between groups (*p* < 0.05); PPO, Peak power output; APO, average power output.*

In the 30-s sprint test, the SST group increased PPO (*p* = 0.001) and APO (*p* = 0.001) from pre- to post test, while no differences were seen in the HST group in PPO (*p* = 0.065) or APO (*p* = 0.057). In addition, a significant differences were seen in change between groups from pre- to post-test in both in PPO (*p* = 0.015, ES = 1.00) and APO (*p* = 0.032, ES = 0.85).

In the 55-m sprint test performed in a seated position, the SST group showed a significant improvement from pre- to post test (*p* < 0.001), while no significant change was observed when the test was performed in a standing positon (*p* = 0.330). No significant changes were observed in the HST group in seated position (*p* = 0.734) or in standing position (*p* = 0.752) In addition, a significant difference was observed between the groups in change from pre- to post test in the seated position test (*p* < 0.001, ES = 1.97) (Table 2). A correlation was also seen in the SST group between improvement in APO in the 6-s sprint test in fresh state and the 55-m seated sprint (*r* = −0.74, *p* = 0.036).

**TABLE 2:** Physiological variables measured pre and post 6-weeks of short-sprint training (SST) or heavy strength training (HST).

	**SST group**			**HST group**		
	**pre**	**post**	**% change**	**pre**	**post**	**% change**
Body mass (kg)	80.1 ± 8.7	79.6 ± 9.1	−0.7 ± 1.4	78.0 ± 9.9	78.6 ± 9.8	−0.3 ± 1.0
Body fat (%)	12.2 ± 4.5	12.1 ± 4.1	−0.1 ± 1.0 p	12.9 ± 5.8	12.4 ± 5.7	0.5 ± 1.6^p^
SMM (kg)	40.2 ± 5.2	40.0 ± 5.2	−0.6 ± 2.0	39.0 ± 4.8	39.3 ± 5.0	0.7 ± 2.5
** 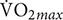 ** test:						
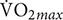 (L min^–1^)	5.03 ± 0.60	5.11 ± 0.65	1.6 ± 4.7	4.80 ± 0.66	4.95 ± 0.59^∗^	3.4 ± 5.2
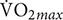 (mL⋅min^–1^⋅kg^–1^)	63.3 ± 5.9	64.8 ± 6.2	2.5 ± 4.3	61.1 ± 6.0	63.4 ± 6.7^∗^	3.8 ± 5.5
W_*max*_ (W⋅kg^–1^)	5.2 ± 0.5	5.4 ± 0.5^∗^	3.2 ± 2.8	5.2 ± 0.6	5.2 ± 0.6	0.6 ± 2.9^#^
**4 mmol⋅L^–1^ [La^–^]**						
Power (W⋅kg^–1^)	3.7 ± 0.5	3.8 ± 0.5	2.3 ± 4.9	3.6 ± 0.5	3.7 ± 0.5^∗^	4.3 ± 5.6
**Gross Efficiency (%)**						
Fresh state (%)	20.3 ± 1.3	20.0 ± 1.1	−0.3 ± 0.9p	20.5 ± 1.0	20.2 ± 1.1	−0.3 ± 0.5*p*
Prolonged cycling (%)	19.7 ± 0.6	19.7 ± 0.8	0.1 ± 0.7p	19.5 ± 1.0	19.5 ± 0.7	0.0 ± 0.7^p^

*Values are mean ± SD. ^∗^Difference within-groups (*p* < 0.05); ^#^Difference between groups in change (*p* < 0.05); SMM; sum muscle mass, RER, respiratory exchange ratio; VE, ventilation; HR_*max*_, maximal heart rate; W_*max*_, maximal aerobic power; [La^–^], blood lactate concentration; Gross Efficiency, at 2 mmol⋅L^–1^ [La^–^]; p, percentage point change.*

### 5-min All-Out Performance

Both the SST and HST group increased APO from pre- to post test in 5 min all out performance (*p* = 0.001 and *p* = 0.004, respectively) (Table 1), with no significant difference in change between groups from pre- to post test (*p* = 0.273, ES = 0.0.43).

### 1RM-Squat and Body Composition

From pre- to post test, the HST group increased load in the 1RM half-squat test by 9.1 ± 3.7%, from 125 ± 24 kg to 136 ± 25 kg (*p* < 0.001), while the SST group showed a 3.9 ± 3.8% decrease, from 128 ± 32 kg to 122 ± 30 kg (*p* = 0.001). In addition, a significant difference in change between the groups was observed (*p* < 0.001, ES = 3.40).

No difference within groups was found in body mass (kg) (SST: *p* = 0.055, HST: *p* = 0.339), body fat (%) (SST: *p* = 0.856, HST: *p* = 0.157) or muscle mass (kg) (SMM) (SST; *p* = 0.302, HST; *p* = 0.215). Nor was a significant difference in change seen between groups in body mass (*p* = 0.364, ES = 0.34), body fat (*p* = 0.398, ES = 0.33) or SMM (*p* = 0.118, ES = 0.61) (Table 2).

### V̇O_2__*max*_, W_*max*_, Power Output at [La^–^] of 4 mmol⋅L^–1^, and Gross Efficiency

In 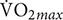, the HST group had a significant increase from pre- to post test (*p* = 0.012). No significant changes were observed in the SST group (*p* = 0.103). However, there were no group differences in changes between groups (*p* = 0.502, ES = 0.26) (Table 2).

In W_*max*_, the SST group had a significant increase from pre- to post test (*p* = 0.002), whereas no change was observed in the HST group (*p* = 0.580). In addition, a significant difference in change between groups was observed (*p* = 0.037, ES = 0.91).

At [La^–^] of 4 mmol⋅L^–1^, the HST group significantly increased power output from pre- to post test (*p* = 0.004). No significant change was observed in the SST group (*p* = 0.060). However, no significant difference in change between groups was observed (*p* = 0.345, ES = 0.37).

In GE fresh state and GE after prolonged cycling, there was no significant difference from pre-to post test in neither of the groups (all *p* > 0.158) (Table 2). In addition, no significant difference in change between groups was observed both in fresh state and after prolonged cycling (*p* = 0.685, ES = 0.15 and *p* = 0.891, ES = 0.05, respectively), (Table 2).

## Discussion

The primary aim of the present study was to compare the effects of HST and SST following a 4-week preparation strength-training period on sprint and endurance capacities in trained cyclists. The main findings showed that: (1) 6 weeks of SST induced greater improvement in PPO and APO in 6-s sprint performance both in fresh state and after prolonged cycling, as well as greater improvement in PPO and APO during a 30-s sprint test compared to HST. (2) SST also led to greater improvement than HST in 55-m sprint performance on the cyclists’ own bike, with a significant correlation between the improvement in the 6-s sprint performance and gains in the 55-m seated sprint within the SST group. (3) HST induced a larger 1RM improvement than SST in the half-squat test. (4) No group differences were detected in the 5-min all-out test, 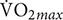, GE, or power output at 4 mmol⋅L^–1^ [La^–^], while the SST group increased W_*max*_ more than the HST group.

### Sprint Performance

Although both SST and HST are common training methods that intend to improve sprint performance in competing cyclists, this is the first study to compare the effects of these types of training on various factors ranging from sprint to endurance indices and maximal strength. A main finding in the present study was the significantly greater improvement in sprint performance in the SST group compared to the HST group. The positive effects of SST on sprint performance were expected, since previous studies conducted both on untrained (Linossier et al., 1993, 1997) and relatively trained (Jakeman et al., 2012) participants showed similar results. Although improvement after SST is previously shown, this is the first study to show that SST is more effective than continuing HST on the aforementioned sprint performance measures in trained cyclists. The specificity of the SST is the most likely explanation for the greater improvement in the SST group. However, the greater metabolic disturbance caused by SST (Kristoffersen et al., 2018) might be an additional reason for the greater improvement in APO in the 30-s test, where lactic anaerobic capacity is needed (Jacobs et al., 1983; Ross and Leveritt, 2001).

In contrast to our findings, previous studies report increased PPO and APO in well-trained cyclists in 30-s all-out cycling after 10–12 weeks of HST (Ronnestad et al., 2010a, 2016). The lack of improvement in our study might be explained by the shorter intervention period, or by the 4-week preparation strength-training period performed by all participants before the start of the 6-week intervention period. The 4-week strength-training period was added in order to reflect common practice in the competitive cyclists’ preparatory period. During this 4-week period, increased PPO and APO were seen in the 6-s sprint test in fresh state and in PPO during the 30-s test, indicating that a short duration of strength-training may improve short-sprint performance in trained cyclists. However, sprint performance during the following 6-week intervention did not improve after HST, although this group improved 1RM in half squat during the same period. Therefore, it is likely that the neuromuscular adaptations relevant to sprinting faster were taken out in the first few weeks with preparatory strength-training, before more specific stimulation through SST was needed to gain further effects on sprinting. The specific effects of HST led solely to higher 1RM strength, without any indications of hypertrophic response. Muscle hypertrophy has been related to improved cycling sprint performance after HST (Ronnestad et al., 2010a; Vikmoen et al., 2016) and the present 6 weeks intervention may have been too short to achieve muscle hypertrophy, at least when combined with a relatively large amount of endurance training (Kraemer et al., 1995). In the future, it should be investigated whether a longer period of SST would further accelerate the effect on sprint performance indices, or if an initial focus on HST, followed by a change in stimulus through more explosive and movement-specific exercise (as experienced by the SST group) is more beneficial. This mix of HST and SST training is indeed interesting, as our findings showed a 3.7% decrease in strength after 6 weeks of SST, indicating that SST does not satisfactorily maintain any HST-induced strength gain. Therefore, long periods without strength training maintenance should be avoided if cyclists wish to maintain or develop their strength (Ronnestad et al., 2015b).

In this study, we also examined whether SST performed on an ergometer and HST could improve sprint performance on the cyclists’ own bike. In previous studies, the association between ergometer cycling and outdoor cycling is not clear, and it has even been suggested that sprint tests should be performed in actual cycling to provide valid assessment of actual changes in performance (Bertucci et al., 2005). In the present study, we showed that 6 weeks of SST on an ergometer increased 55-m sprint performance in actual cycling, while no improvement was seen after HST. Furthermore, we showed strong significant associations between the changes in APO in the 6-s sprint test on the ergometer and in the 55-m seated sprint on the cyclists’ own bike in the SST group. This indicate that 6 weeks of SST on an ergometer have great ecological validity for improvement in the field. In contrast, no change was seen when the 55-m sprint was performed standing, confirming the specific significance.

### Endurance Performance and Physiological Capacities

In the 5-min all-out test, the SST and the HST showed significant increases in APO by 5.7 and 3.3%, respectively, with no significant differences between groups. The 5-min all-out test was chosen as a functional measure of the capacity for very intensive cycling (Hansen et al., 2006; Ronnestad et al., 2011; Vikmoen et al., 2016), with both aerobic and anaerobic contribution. No previous studies have examined the effects of SST on such tests, while improvement has been reported after HST (Ronnestad et al., 2011; Vikmoen et al., 2016). The direct mechanisms behind this study’s 5-min APO improvement after SST remain unclear. However, it is well known that adding HST to endurance training can improve endurance performance (Hausswirth et al., 2010; Koninckx et al., 2010; Ronnestad et al., 2010a; Sunde et al., 2010; Aagaard et al., 2011). Increased leg strength may increase peak torque in the pedal stroke, reduce time to peak torque, and reduce the pedaling torque relative to maximal strength (Ronnestad et al., 2015a). This may allow for higher power output and/or increased blood flow, and in turn improve cycling performance (Ronnestad et al., 2011; Barrett-O’Keefe et al., 2012). In addition, strength training may potentially increase the buffer capacity via increased muscle mass, which in turn will lead to improved anaerobic capacity. However, in the present study, neither of the groups increased muscle mass or improved GE. However, a tendency for an increase in VO_2__*peak*_, coinciding with greater training volume throughout the intervention period in both groups, is a more likely explanation for the improvements in 5-min performance found here.

While no differences between the groups were found in 5-min all-out performance, a significant difference between groups was seen in change from pre- to post-test in W_*max*_ with a significantly greater improvement in the SST group. The W_*max*_ tests demand more high-power production, and since the SST group also showed superior improvements in 6 and 30-s performances, neuromuscular characteristics related to high-power production might have contributed. In contrast to our findings, HST has previously proven to also increase W_*max*_ or time to exhaustion at W_*max*_ in cyclists (Hickson et al., 1988; Ronnestad et al., 2010a; Sunde et al., 2010). However, in agreement with our findings, this positive effect was not observed in a previous study with 6 weeks of strength training (Levin et al., 2009). Unfortunately, we did not have a control group, and cannot conclude whether adding HST or SST has a better effect on 5-min all-out cycling than endurance training alone.

Previous studies have reported GE both as unaffected (Aagaard et al., 2011; Ronnestad et al., 2016), reduced (Aagaard et al., 2011) and improved (Sunde et al., 2010; Ronnestad et al., 2011) after HST in cyclists. To the best of our knowledge, no previous studies have investigated how SST affects GE. However, our results showed no change in GE after 6 weeks of SST or HST, or difference in change between groups, neither in a fresh state nor after prolonged cycling.

The present study was conducted during the preparatory period, and as expected, both groups had increased endurance training volume during this period. After 6 week, the HST group had a significant increase in 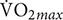 and power output at [La^–^] 4 mmol⋅L^–1^, which is in line with previous findings, and indicating that HST don’t have any negative interference effects on the endurance capacity (Ronnestad et al., 2010a). However, there were no differences between the groups in 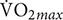, power output at [La^–^] 4 mmol⋅L^–1^ or total training volume after 6 weeks of SST and HST. Therefore, it is likely that the increase in 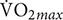 is caused by changes in endurance training in this period, and not the 6-week intervention of SST or HST.

## Conclusion

Short-sprint training caused a larger increase in PPO and APO during 6- and 30-s all-out cycling on the ergometer, as well as in 55-m sprints on cyclists’ own bikes compared to the corresponding effects of HST in trained cyclists. In comparison, HST led to a larger increase in maximal strength than SST, while no group differences were found in 5-min all-out performance, 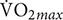, power output at 4 mmol⋅L^–1^ [La^–^], or gross efficiency. Altogether, this shows a high degree of specificity of the implementation of both SST and HST, with SST providing superior speed and power gains whereas HST are most effective for improving strength, and no differences in their influence on endurance variables among trained cyclists during a 6-week intervention period. SST performed on an ergometer also seems to transfer to improved sprint performances on the cyclist’s own bike, which indicates that SST performed on an ergometer is a valid training method for road cyclists.

## Data Availability

The datasets generated for this study are available on request to the corresponding author.

## Ethics Statement

Human Subject Research: The studies involving human participants were reviewed and approved by the Norwegian Centre for Research Data (NSD). The patients/participants provided their written informed consent to participate in this study.

## Author Contributions

MK: planning, data collection, statistical analysis, and writing. ØS and BR: planning and writing. HG: planning, statistical analysis, and writing.

## Conflict of Interest Statement

The authors declare that the research was conducted in the absence of any commercial or financial relationships that could be construed as a potential conflict of interest.
